# Intake of nanoparticles and impact on gut microbiota: *in vitro* and animal models available for testing

**DOI:** 10.1017/gmb.2021.5

**Published:** 2021-12-28

**Authors:** Débora Campos, Ricardo Goméz-García, Diana Oliveira, Ana Raquel Madureira

**Affiliations:** 1 CBQF—Centro de Biotecnologia e Química Fina, Laboratório Associado, Escola Superior de Biotecnologia, Universidade Católica Portuguesa, Porto, Portugal; 2 Amyris Bio Products Portugal, Unipessoal Lda, Porto, Portugal

**Keywords:** Microbiota, Gut, Nanoparticles, Models

## Abstract

The oral delivery of compounds associated with diet or medication have an impact on the gut microbiota balance, which in turn, influences the physiologic process. Several reports have shown significant advances in clarifying the impact, interactions and outcomes of oral intake of nanoparticles and the human gut. These interactions may affect the bioavailability of the delivered compounds. In addition, there is a considerable breakthrough in the development of antimicrobial nanoparticles for intestinal pathogenic bacteria. Several *in vitro* fermentation and *in vivo* models have been developed throughout the years and were used to test these systems. The methodologies and studies carried out so far on the modulation of human and animal gut microbiome by oral delivery nanosized materials were reviewed. Overall, the available *in vitro* studies mimic the real physiological events enabling to select the best production conditions of nanoparticulate systems in a preliminary stage of research. On the other hand, animal studies can be used to access the dosage effect, safety and correlation between haematological, biochemical and symptoms, with gut microbiota groups and metabolites.

## Human gut microbiota

Human’s small intestine, comprising the duodenum, jejunum and ileum, is the site where all food compounds, previously digested in the mouth and stomach, arrive to be further digested and majorly absorbed. Water and electrolytes uptake occurs further down in the colon, as well as fermentation of polysaccharides and proteins by colonic microbiota, re-absorption of bile salts and elimination of faeces (Holscher, 2017; Gutiérrez-Sarmiento et al., 2020). Colon is the section of the large intestine greatly colonised by bacteria, fungi, viruses and Archaea, forming a complex ecosystem called gut microbiota (Flint and Juge, 2015). This microbiota lives in a close relationship with the host and has a great influence on the host’s health. More recently, the interaction between the gut and brain, known as the gut–brain axis, has been identified, due to metabolic signals. The gut–brain axis is a bidirectional communication network between the central nervous system and the gastrointestinal tract (GIT), which constitutes part of the nervous system (Javed et al., 2020a). The entire human GIT is populated by *ca.* 100 trillion bacteria, representing up to a thousand different genera and species. The bacterial numbers rise from duodenum (10^2^ bacteria/g) to colon (10^12^ bacteria/g), and the total weight of bacteria can attain *ca.* 1 kg in an average adult, comprising of up to 5,000 species (de Carvalho et al., 2019). Throughout the digestive tract, the composition and numbers of these communities vary, and every individual has their specie collection, which in turn leads to adjustments over lifetime and is affected by the host lifestyle and nutritional diet (Madureira and Pintado, 2018).

Most of the human microbiome is innocuous or beneficial to the host and act as a protector against pathogens, providing nutrients and energy, and fosters development (Fu et al., 2019; Hasan and Yang, 2019). Typically, there is a prevalence of bacteria belonging to the phyla Bacteroidetes and Firmicutes. *Lactobacillus* and *Bifidobacterium* species belong to Firmicutes and Actinobacteria divisions, respectively and are considered as the beneficial gut bacteria associated with the control of GIT functions, such as regulation of intestinal transit and inhibition of potential pathogenic bacteria such as *Salmonella* spp. and *Escherichia coli*, e.g. through the production of inhibitory organic acids. Besides the functional roles in normal digestion, these bacteria also play several immunological roles, namely conjugation of bile acids; prevention of pathogenic bacteria growth; production of butyrate that regulates colonic enterocyte health; production of vitamins B12 and K; detoxification (or toxification) of certain ingested drugs or plant toxins; immune system maturation and modulation of the metabolic pathways with different organs in the human body (Koppel et al., 2017; Scher et al., 2020). Nevertheless, there are host–bacteria interactions specific differences occurring at the genus level, belonging to Proteobacteria, Actinobacteria, Verrucomicrobia, Cyanobacteria and Deferribacteres groups and Fusobacteria. Pathogenic microbiota can also be found and pathobiont causing disease and intestinal homeostasis, and the molecular mechanisms by which pathobionts causes disease remains poorly understood (Madureira and Pintado, 2018).

Daily, gut suffers unmanageable changes, as reduction of gut oxygen content and is affected by several external factors such as environment pollution and antibiotics intake, which may cause microbial imbalance (dysbiosis), increasing the susceptibility to diseases and ultimately lead to an unhealthy state sometimes difficult to revert or rectify (Fu et al., 2019). Obesity, inflammatory bowel disease, intestinal infections and cancerous lesions of the intestine, liver and pancreas, autoimmune diseases, and many behavioural and psychiatric issues have been associated to a disordered and impaired microbiota community (Naseribafrouei et al., 2014; de Carvalho et al., 2020). Thus, diet is aspect key factor when comes to control the abundance of human gut microbes, their health state and balance and their metabolites, such as short chain fatty acids, production, well acknowledged for their health benefits. Therefore, the increasing demand for natural, safe and clean label foodstuffs/ingredients, with beneficial and health-promoting characteristics, has contributed to diverse research studies for the development of novel functional food ingredients rich in bioactive compounds. Polyphenols, e.g. have been shown to positively modulate gut microbiota, with especial impact on *Bifidobacterium* and *Lactobacillus* bacteria (Campos et al., 2020), (Ribeiro et al., 2021). However, these compounds are susceptible to extensive loss or degradation and modifications in the upper GIT after ingestion, which consequently reduce their final concentration, affecting their functionality and activity in the gut. In this regard, different oral-safe protective systems, such as nanoparticles (NPs) have been used to overcome these issues and then exert efficiently their beneficial outcomes (Madureira, Pereira, & Pintado, 2015; Madureira et al., 2016; Tang et al., 2020). Studies and recent trends on digestion of these NPs and the impact of GIT conditions in the stability, loaded compounds release and bioavailability are discussed in this review. In addition, the interactions with the gut microbiota will also have an important and decisive role on the metabolism of these NPs. These bacteria can metabolise these nano-systems and affect their adsorption by the intestinal epithelium, or on the other hand, these NPs can change the microbiota positively or negatively originating other physiological states. Thus, there is a need for targeted toxicological investigations on the influence of ingested compounds, such as NPs, on the gut microbiota.

## NPs digestion

There is a great number of studies on digestion and gut microbiota modulation by NPs. Most of the studies found use metal NPs, such as silver and copper NPs, as shown in Table 1, but there are also studies using carbon nanotubes (CNT) and solid lipid nanoparticles (SLN). In this article, most of the studies discussed include silver NPs (AgNPs), silica (SiO_2_), titanium (TiO_2_) and zinc (ZnO) NPs, as these are commonly added as ingredients to foods and health care products. TiO_2_, ZnO and SiO_2_ are produced in the highest amounts, while AgNPs are used in a higher number of products.

**Table 1.** Studies available on the effects of NPs on gut microbiota.

Model	Nanoparticles	Microbiota effects/interactions	Target microbiota	Reference
*In vitro* (human faecal material)	AgNPs	Antimicrobial effects/growth promoter	Bacterial isolates, Bacteroides, *Roseburia* spp.*/Escherichia coli*, *Raoutella* spp.	(Taylor et al., 2015b)
		Changes in numbers (1 μg/ml)	Firmicutes (increase) and Bacteroidetes (decrease)	(Cattò et al., 2019)
	Zinc, cerium and TiO_2_ NPs	Phenotypic changes	General groups (e.g. Firmicutes, Bacteroidetes, Bacteroides and Universal)	(Han et al., 2010b)
	SLN	Antimicrobial effects (empty SLN)	General groups (e.g. Firmicutes, Bacteroidetes, Bacteroides, Universal)	(Wang et al., 2011b)
		Growth promoter (loaded with herbal extracts)	*Bifidobacterium* spp., *Lactobacillus* spp.	
*In vitro* (animal faecal material)	Selenium-NP	Prebiotic and antimicrobial	*E. cecorum*	
*In vivo* (mice and rats)	Copper-loaded chitosan NPs	Antimicrobial effects	Coliforms	(Merrifield et al., 2013b)
	AgNPs	No effect detected	n.a.	(Hadrup et al., 2012b); (Wilding et al., 2016b)
		Antimicrobial effects	Firmicutes, *Lactobacillus* spp.	(Williams et al., 2015b)
		Antimicrobial effect depending on NPs shape (0.2 mg/ml)	*Clostridium* spp., *Bacteroides uniformis*, Christensenellaceae and *Coprococcus eutactus*, *Oscillospira* spp., *Dehalobacterium* spp., Peptococcaeceae, *Corynebacterium* spp. and *Aggregatibacter pneumotropica*	(Javurek et al., 2017)
	Silica and AgNPs	Population numbers of changes	Bacteroides, Firmicutes and Actinobacteria	(Lecloux et al., 2015)
	AgNPs (3 mg/kg BW)	Population numbers of changes linked to behavioural and metabolic alterations	*Coprobacillus* spp., *Mucispirillum* spp., and *Bifidobacterium* spp. reduced, *Prevotella* spp., *Bacillus* spp., *Planococcaceae*, *Staphylococcus* spp., *Enterococcus* spp. and *Ruminococcus* spp. increased	Zhen lyu
	TiO_2_ NPs (5 mg/ml)	Alteration of gut microbiota during pregnancy and increased the fasting blood glucose of pregnant rats	General groups (e.g. Firmicutes, Bacteroidetes, Bacteroides and Universal)	(Mao et al., n.d.)
	TiO_2_ NPs, SiO_2_ NPs and AgNPs (2.5 mg/kg BW/day)	Changes in groups and numbers	Bacteroidetes and Firmicutes (shifts in the intra- and inter-phyla abundance); *Lactobacillus* (decrease)	(Chen et al., 2017)
	SLN (acute)	Dose and lipid related changes	Firmicutes, Bacteroidetes	(Madureira, Nunes, et al., 2016)
	SLN (chronic)	Growth promoter (loaded with herbal extracts)	Firmicutes, *Lactobacillus* spp.	(Madureira et al., unpublished)
	Mesoporous silica (MSNs) MCM-41, SBA-15 and DMSN	Toxicity	*Verrucomicrobia* (decrease)/*Candidatus Saccharibacteria* in MCM-41 (increase)	(Yu et al., 2021)
*In vivo* (fish)	Copper loaded NPs and AgNPs	Antimicrobial effects	*Cobacterium somerae*	(Sawosz et al., 2007b)
	*Shewanella putrefaciens* Pdp11	Growth promoter	Lactic acid bacteria	(Cordero et al., 2015b)
		Changes in microbiota groups	Proteobacteria, Cyanobacteria and Vibrio	
Male zebrafish	AgNPs	Dysbiosis	General groups (e.g. Firmicutes, Bacteroidetes, Bacteroides and Universal)	(Ma et al., 2018)
*In vivo* (avian species)	Copper-loaded chitosan NPs	Antimicrobial effects	*Escherichia coli*	(Wang et al., 2015b)
		Growth promoter	*Bifidobacterium, Lactobacillus* spp.	
	AgNPs	Growth promoter	Lactic acid bacteria (except *Lactobacillus*)	Stilling et al. (2014)
*In vivo* (pigs)	AgNPs	Antimicrobial effects	Firmicutes, *Escherichia coli*	(Bellmann et al., 2015c)
	ZnO NPs (Nano-ZnO) 600 mg Zn/kg	Changes in growth and diversity	*Streptococcus*, *Lactobacillus* (increase) in ileum; *Lactobacillus*, *Oscillospira* and *Prevotella* (decrease) in colon	(Xia et al., 2017)

Mobil Composition of Matter No. 41 type mesoporous silica (MCM-41) group; Santa Barbara Amorphous-15 type mesoporous silica (SBA-15).

Abbreviations: NPs, nanoparticles; SLN, solid lipid nanoparticles.

### Oral ingestion and digestion of nanoparticles

Before the studies involving the gut microbiota, it is important to evaluate the bio accessibility and bioavailability of NPs, to understand their path along all GIT, from mouth to colon in order to comprehend the chemical changes that occur during digestion. After oral ingestion, there are mechanical forces and significant pH changes along the GIT that need to be considered. The physical (contractions, peristaltic movements, temperature, mucus viscosity and interfacial interactions) and chemical (pH, enzymes and mucus composition) parameters may affect the NPs size and surface properties. In the stomach, high energetic contractions have been measured, but effects on NPs agglomeration and aggregation are still unknown (Bellmann et al., 2015a). The changes of pH along the GIT occur mainly during the fasted state, however pH is usually buffered to a range of 2–6 in the presence of food. Low pH can increase dissolution of particles and enzymes in the digestive fluids can induce particles denudation.

**Figure 1:**
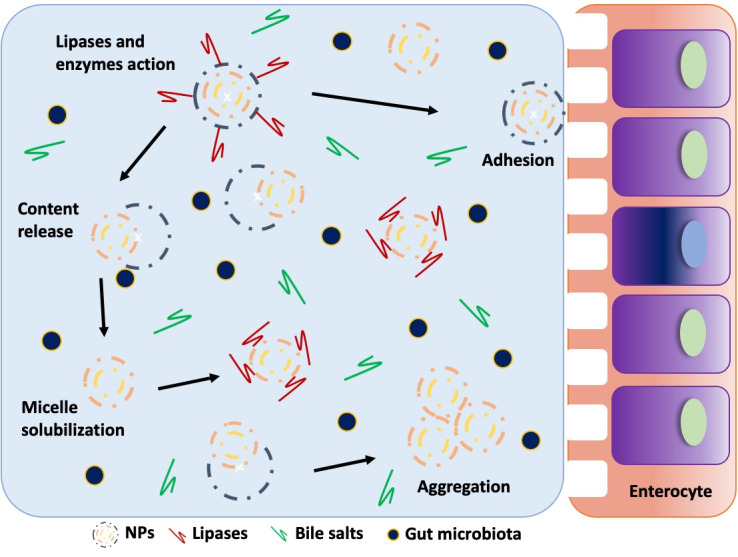
Hypothetical faith of solid lipid nanoparticles (SLN) when reaching the small intestine. The behaviours of solubilization, content release and aggregation are shown, which typically occurs upon this type of nanoparticles once reaching the small intestine.

The different steps throughout digestion promote chemical and physical changes of the NPs. The simulation of GIT’s conditions has been carried out in many ways during the last two decades, including by our research team, who developed one of the first GIT models that performed exposition of food samples to the different steps of digestion (Madureira et al., 2005; Madureira et al., 2011). Nevertheless, owing to the number of models for simulation of GIT conditions, an European COST action project has standardised an unique human model (INFOGEST protocol) to be used among the scientific community (Minekus et al., 1999; Cinquin et al., 2006; Brodkorb et al., 2019). Several studies have simulated the dynamic passages of NPs through the GIT using artificial saliva (pH 6.8 and porcine/human α-amylase), gastric (pH 1.3 and porcine pepsin) and intestinal juices (pH ~ 8, with duodenal and bile juice – bile salts and porcine pancreatin). The path of NPs along GIT is strongly influenced by their composition and size. Generally, the mouth digestion does not have an impact upon the NPs, but the same is not reported for the following steps. The gastric fluids at very acid pH and high electrolyte concentrations, lead to a high level of agglomeration. However, in the following step and due to the intestinal juice, pH increment and enzymes chemical digestion, the NPs deagglomerate. These effects were especially observed with AgNP, in which the gastric fluid provoked partial dissolution and release of Ag+ (Walczak et al., 2012; Bellmann et al., 2015b). Solid lipid nanoparticles were also shown to be considerably affected by the intestinal juice (pancreatin and bile salts), suffering dissolution followed by aggregation (Madureira, et al., 2016). The mechanistic behaviour of the interactions with NPs and digestion are demonstrated in the Figure 1, such as the dissolution, aggregation and absorption of nanoparticles and bioactive molecules. Serini et al. (2018) used resveratrol-based SLNs loaded with Omega-3 PUFA and showed that oxidation and degradation processes were achieved along digestion enhancing their antineoplastic activity. Also, nanochitin demonstrated to retard lipid digestion by promoting aggregation of the oil droplets under INFOGEST simulated gastrointestinal conditions. Thus, it reduced the final extent of lipid digestion in the small intestine, as well as decreased the bio accessibility of the encapsulated carotenoids. The same trend was observed by Afonso et al. (2020), and concluded that zein NPs are better carriers of β-carotene than ethyl cellulose, for crossing GIT conditions. Thus, the compounds used to produce the particles have huge impact in their stability, dictating their successful bioavailability in the intestinal phase. It is evident that digestion stages greatly influence the stability of NPs, and the compounds used for the development of these systems must consider their resistance to gastric fluids in addition to the agglomeration events, which can compromise the bioavailability of these particles when reaching the intestine. The models to simulate this stage are very useful to access the NPs material stability throughout digestion and do not compromise the delivery of the encapsulated compounds in the intestine. Presently, the best model available to test the chemical and biological stability of these systems is the INFOGEST protocol, which allows controlling the conditions in all stages and is a uniformed model used by several research groups. This protocol was designed to be carried out using standard laboratory equipment and includes a static digestion method that uses constant ratios of meal to digestive fluids and a constant pH for each step of digestion.

**Figure 2:**
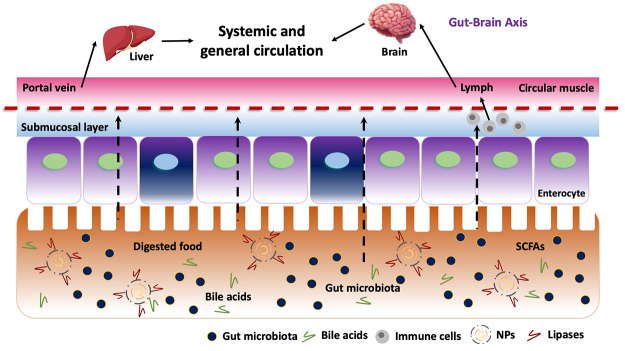
Impact of nanoparticles when reaching the intestinal digestion and hypothetical pathways until the liver and brain and indirect impact at the immunological and metabolic interactions (and gut–brain axis).

When reaching the intestinal epithelium, the presence of mucus influences the contact of NPs with bacteria in the lumen. Figure 2 shows an example of the gastrointestinal roadmap digestion of NPs (e.g. SLN) when reaching the intestinal phase. The epithelial cells are coated by a mucus layer that comprises a firmly and loosely adherent layer that can reach a total thickness of up to 1,000 μm, producing a strong barrier that prevents diffusion of both bacteria and NPs in cells. This restriction is made by attachment of mucus fibres through ionic and hydrophobic interactions and by size filtering (Fröhlich and Roblegg, 2012; Froehlich and Roblegg, 2014).

Another emerging topic on the area of NPs intake is associated with the passage of NPs from the intestinal lumen to the blood stream and their interaction with biological fluids, specifically with proteins, forming at the NPs surface, a layer called ‘protein corona’. This structure is personalised from donor to donor, since the surface proteins might vary due to changes associated with the plasma donor. This characteristic allows identifying a corona fingerprint of distinct plasma donor, with respect to protein composition and quantities. The identified proteins are linked with physiological functions, such as, immunoglobulin (~80% of the total protein) with complement and coagulation. Other proteins found were responsible for tissue leakage, acute phase and apolipoprotein. In addition, the description of the most common protein part of the corona, enables to determine the potential of NPs where the main type of proteins are immunoglobulins with a critical role in immune responses (Ren et al., 2019).

## NPs and gut modulation

The interaction between NPs and the gut microbiome plays a key role in metabolism, food digestion, pathogen clearance and active nutritive connection with other organs, such as the known gut-brain axis. Javed et al. (2020) have provided an overlook on the implications of possible changes of the human gut. First affecting the nutrients digestion, absorption and distribution, influencing the active involvement of the gut with neuronal innervation, blood circulation and immune system and secondly, all these implications communicate with other organs from the human body.

The gut microbiota is continuously changing by the influence of lifestyle, eat habits, medicines and environmental changes that human body undergoes throughout lifetime. These changes have been reported to have negative and positive impact on the human behaviour and consequently in health. Thus, studies concerning the interaction of different types of NPs with gut have been carried out to understand their impact and how to manage their benefits towards the promotion of human well-being and health.

The next sections will focus in the available *in vitro* and *in vivo* models and on what can be done to mimic *in vitro* the real conditions of colon. In addition, the advantages of using them and, examples of the research work with these models and also information that  animal models can give when working with NPs is discussed.

### In vitro studies with gut microbiota

There are several types of studies using gut microbiota, available in literature. Most of them use monocultures isolated from gut and follow the direct effect of NPs in the growth and metabolism of these specific bacteria. In these studies, culture synthetic media and anaerobic conditions of incubation are normally used to mimic intestinal conditions. Nevertheless, when studying prebiotic activities there are some authors that only focus on lactic acid bacteria, known as probiotic, such as those belonging to *Lactobacillus* and *Bifidobacterium* genus. Others use pathogenic intestinal bacteria, looking for the antimicrobial effect of NPs and predicting their use as ingredients for control of infectious intestinal diseases.

When performing these studies, independently of their format and complexity, the optimal growth conditions of each bacteria genus should be considered, since most of the gut microorganisms are restrictive fastidious anaerobes. *In vitro* studies can also use faeces from human volunteers as inoculum media, owing to the richness and real representation of the gut microbiota. In this case, faecal samples must be exclusively manipulated in an anaerobic chamber to mimic colon atmosphere (Madureira et al., 2016). The manipulation of these strains or material rich in anaerobic bacteria, such as faeces, involves the use of specific atmosphere environment conditions (5% H_2_, 10% CO_2_ and 85% N_2_) and a specific basal media simulating the entire chemical and nutrients to maintain alive the million different strains present. Faeces are diluted in such media and the anaerobic conditions inside the reactors must be guaranteed using a pH indicator dye (e.g. resazurin). The NPs are further incorporated and incubated at 37°C and the bacterial growth and metabolic pattern can be evaluated during incubation time. Most of the studies use fresh stool samples; nevertheless, the availability of fresh faecal inoculum and its inherent variability is often a problem. Moreover, the use of faecal inoculums for *in vitro* fermentation models requires a viable gut microbiota, capable of fermenting the unabsorbed nutrients. Hence, our research team recently developed a method that allows to preserve samples using phosphate-buffered saline and 30% glycerol solution to maintain the gut microbiota viability during storage at −20°C for at least 3 months, without interfering with the normal course of colonic fermentation (de Carvalho et al., 2021).

In practice, the fermentation trials with human faeces include dispersing the donor’s faecal material in batch-controlled reactors/vessels containing basal medium but can also be dynamic. Batch systems use small reactor vessels or test tubes mimicking only one single segment of the GIT, and each vessel is connected to an anaerobic mixture gas supplier, a pH controller and has the temperature regulated by a water bath. In the case of studies with NPs, these should be used preferentially dried so they can be mixed with the inoculum and incubated at their optimal conditions. Dynamic models represent better the physiological conditions occurring in the gut, and in this class we can find SHIME, EnteroMix, the Lacroix model and TIM-2, which differently from batch, are composed by two or more chambers connected by vessels or membranes that simulate how the lower or complete digestive tract allows the continuous flux of fluids (Minekus et al., 1999; Cinquin et al., 2006; Makivuokko et al., 2009). Different types of NPs have been already tested in this type of model using human faeces. Besides having an idea of the impact of NPs on the overall groups comprising the gut microbiota, it is also possible to withdraw conclusions regarding the impact in their diversity since the identification and quantification is mostly done using quantitative polymerase chain reaction (qPCR) or Next-Generation Sequencing (NGS). During incubation time, the growth profiles and metabolism can be followed, and the NPs interaction with the main microbiota groups observed.

The first approach of several research works is to test the effect of NPs in microorganisms isolated or typical species of the gut microbiome. Most of the times, the microorganisms tested are representative of the beneficial bacteria, such as probiotic; however, other such as pathogenic bacteria responsible for infectious diseases can also be studied. As example, our research team evaluated the effect of chitosan NPs loaded with rosmarinic acid, protocatechuic acid and the 2,5-dihydroxybenzoic acid in *Bacillus cereus*, *E. coli* O157, *Listeria innocua*, *S. aureus*, *S. typhimurium* and *Yersinia enterocolitica*, as a representative bacterial group of intestinal infectious diseases (Madureira et al., 2015; Madureira, Pereira, & Pintado, 2015).

Most of the studies available concluded that the NPs effects on gut microbiota are dependent on the NPs dose and physical properties. This was possible by controlling parameters and testing different factors. As example, CNT, single-walled (SW) and multi-walled (MW), pristine and functionalised and non-functionalised CNTs, short and long, were tested at doses ranging from 10 to 100 μg/L in isolated gut bacteria, such as the *Lactobacillus acidophilus*, *E. coli*, *E. coli K12, E. coli* K12 TG1 (plux), *Bacillus subtilis, Ochrobactrum* sp., *Paracoccus denitrificans, Staphylococcus aureus, Salmonella typhimurium* and *Enterococcus faecalis.* The antibacterial activity showed to be CNT type-dependent, functionalisation, concentration-dependent and the physicochemical properties of CNT such as rigidity, diameter and length were also associated (Maksimova, 2019). The mechanism of action has also been associated to modifications of the bacterial membrane and to the bacteria shape, since rod shaped bacteria were shown to be more resistant than spherical ones to the activity of antimicrobial NPs (Chen et al., 2013).

The toxicity of NPs on microbiota can also be accessed and different formats and concentrations and materials can be directly tested. Our team tested SLNs loaded with herbal extracts using human faeces to evaluate the interaction mechanisms and study SLNs toxicity in the gut microbiota groups (Madureira et al., 2016). Having the possibility of testing various conditions and with the right number of vessels, is possible to compare free extracts with encapsulated ones. In this specific study, SLN slowly released the phenolic compounds at lower concentrations than free extracts, which did not negatively affected the microbiota and allowed their adaptation to the slowly released compounds. On the other hand, free extracts had a negative impact on the gut microbiota groups.

Other effects such as the modulation of metabolic activity, electrophoretic mobility, hydrophobicity, among others, can also be evaluated, a study with ZnO and cerium oxide (CeO_2_) NPs at 0.01 μg/L and 3 mg/L of TiO_2_ NPs showed that these NPs also affected phenotypes and sugar content of the extracellular polymeric substance, indicating changes in the community’s stability. The most active type of NPs was TiO_2_ certainly because of its lack of dissociation and greater stability (Taylor et al., 2015a).

Considering this information, it is clear the importance of studying the interaction and impact of NPs upon the microbiome, as well as the secondary and indirect impact that might produce into the modulation of metabolic activities within the human body. These examples are relevant why studies involving microbiome are fundamental to guarantee the safety of application in food systems and subsequent consumption.

The best *in vitro* model to test NPs effects in gut microbiota is the one using human faeces samples since more representative of the gut microbiota diversity. The use of isolated bacteria only allows to look specifically to a microorganism’s specie or strain, but do not resemble the entire environment in the gut, not only in terms of microorganism’s diversity, but also regarding the metabolites production. Nevertheless, when searching for a specific effect of NPs, or to understand mechanisms of interaction of NPs with microorganism’s cells such as with membrane, the studies with monocultures are also useful.

### Available animal studies

Most of *in vivo* animal studies use rats and mice as models, however other animals like fish, birds and pigs have also been used. This type of studies enable to assess *in vivo* gut microbial alterations, namely, bacteria growth rate and feed conversion. For example, Zebrafish (*Danio rerio*) microbiota has been used as model for the study of microbial communities in vertebrate intestines (Wang et al., 2015a; Chen et al., 2018). In these studies, animal subjects are usually fed acute or chronically with the target NPs formulation. Feeding is commonly given by gavage, using liquid state NPs or the NPs can be incorporated in the daily diet and faecal samples are taken and analysed throughout the study. In addition, it is also possible to study the animal caecum tissues after necropsy and visualise the NPs and analyse bacterial DNA after its extraction and sequencing by PCR real time or other DNA sequencing methods (Madureira and Pintado, 2018).

Further, the primary concern in these studies is the possible toxicity of NPs, if they are absorbed or accumulated in body organs. However, the particles and compounds released before absorption may also have toxic effects and induce changes in the normal microbiota. Additionally, microbiota may also positively interfere on NPs absorption, e.g. Gram-negative bacteria induce adherence of NPs to lipopolysaccharides and enhance their delivery. Finally, luminal NPs may affect gut microbial metabolism and potentially influence nutrient absorption or xenobiotic metabolism (Cattani et al., 2010).

However, due to animal welfare and protection issues, animal studies are currently being avoided and are only used once *in vitro* tests are concluded, namely when safety and toxicity assessment and effective dose estimation of the target compounds originate promising results, which are worth of further investigation, thus diminishing the number of animals sacrificed. Nevertheless, the use of animal models is still very useful for NPs studies, as it provides biochemical data, enables to follow the absorption and secretion paths of the encapsulated compound in the NPs and helps to understand if the NPs are being accumulated in any organ. In addition, allows to collect faecal material and characterise the gut microbiota changes in terms of diversity and metabolic compounds produced throughout the study. Overall, *in vivo* studies enable to study the NPs’ impact on gut microbiota in several ways, either through their antimicrobial activity and/or inhibition of a specific group, their prebiotic activity and/or enhancement of a specific microbiota group or by a non-explained disruption of microbiota. Nevertheless, in some cases, the absence of effects can be also observed. Table 1 describes several examples associated with animal studies where tests involving NPs and gut microbiota were performed, attending different purposes. Moreover, it was identified the group of bacteria that was specifically studied to get a general overview of what has been performed within this type of research.

Several studies have shown that changes of gut microbiota depend on the size and daily dose of NPs feed to the animals. In addition, some NPs can induce intestinal gene expression, which was related with greater proportions of Gram-negative bacteria in gut microbiota (Williams et al., 2015a). Other studies showed the antimicrobial effect of NPs in pathogenic bacteria with consequent increment of the beneficial groups. This was observed for chitosan NPs loaded with copper when feed to Sprague-Dawley rats (Han et al., 2010a), and in avian broilers chickens (Wang et al., 2011a). Here, the suppression of coliforms was a growth promoter of *Lactobacillus* and *Bifidobacterium*, and not a direct prebiotic effect. The disruption of microbiota and consequent disappearance of one specific group was also observed when the same type of NPs and AgNPs were tested in zebrafish microbiota. *C. somerae* (OTUs Z4 and Z6), a common representative of fish intestinal bacteria was totally vanished from microbiota community (Merrifield et al., 2013a). On the other hand, no changes were observed in studies with AgNPs in rats and mice (Hadrup et al., 2012a; Wilding et al., 2016a).

In another perspective, some studies with the same type of NPs showed variations in the animal’s microbiota population dependent on NPs physical and dose. These changes were observed when using different NPs sizes. Small NPs with *ca.* 10 nm induced a decrease in populations of Firmicutes phyla, along with a decrease of the *Lactobacillus* genus. AgNPs with 60–100 nm also reduced coliforms in the gut microbiota (Fondevila et al., 2009). On the other hand, when NPs with sizes between 14 and 110 nm were used, no changes occurred. Hence, size wise, there is no specific trend identified. Nevertheless, the type of animal used in these studies also showed to influence the NPs’ impact. In bigger animals, as weaning pigs, AgNPs with 110 nm, not only caused a weight increase, but also a decrease in Firmicutes. This decrease was observed at the highest concentration (i.e. 36 mg/kg), which shows that the daily NPs’ dosage plays a significant role and needs to be further exploited. Some *in vivo* studies in mice investigated the effect of the ingestion of silica and AgNPs mixed in food for 28 days, at doses relevant for human diet. This study showed a decrease in Bacteroides and an increase in Firmicutes, in the case of AgNPs depending on the dose used. Whereas a dose-dependent decrease in Actinobacteria was observed in those exposed to silica NPs (Lecloux et al., 2015). Japanese quail also received AgNPs in water at doses of 25 mg/Kg and 10 nm in size. Greater proportions of Firmicutes phyla together with an increase in lactic acid bacteria and a decrease in the *Lactobacillus* genus were observed (Sawosz et al., 2007a). Contrarily, when using AgNPs daily dosages of 4.5–10 mg/kg/day, no changes were observed and only doses above 10 mg/kg/day had impact on rat, mice and avian microbiota. The same trend was observed in van der Brule et al. (2015) study [in which no overall toxicity was detected, but AgNP did disturb bacterial evenness (α-diversity) and populations (β-diversity) in a dose-dependent manner. In addition, metabolic and biochemical markers reported were like those observed for metabolic and inflammatory diseases, such as obesity van der Brule et al., 2015].

The administration of high doses of NPs may lead also to pathological changes in the duodenum and colon, such as inflammatory cell infiltration with ulceration. This occurred when 2.5 mg/kg of SWCNTs was acutely administrated, in contrast with 0.05 mg/kg (Chen et al., 2018). The authors suggested that CNTs had ability to start, as well as intensify the intestinal permeability and gut inflammation of the tested mice, due to the deposit of tubular particles in the colon, after oral administration. Hence, there is evidence that higher doses lead to major changes in gut microbiota or to pathological symptoms that can also originate microbiota dysbiosis. If intestinal permeability is compromised, the passage of harmful substances and microorganisms may occur and immune system becomes deregulated, leading to disease. Overall, the exposure to NPs may alter gut microbiome community composition and interfere with the physiological functions of the intestine, including neurotransmission, epithelial permeability, inflammation and oxidative stress, demonstrating the risks associated with continuous contact with such structures and enabling to elucidate the implications of NPs and gut microbiota (Chen et al., 2018).

Other factors can be related with changes of microbiota when NPs are administrated. Nanoparticles can induce symptoms that by themselves provoke microbiota dysbiosis. This was the case of the oral exposure of rats to ZnO NPs, which caused liver injury and behavioural changes in the treated animals. It might be hypothesised that ZnO induced the behavioural effects and consequently affected the gut microbiota (Hsiao et al., 2013; Wang et al., 2015a). The same was reported in the case of colitis associated to alterations in gut microbiota, in mice exposed to ambient particulate matter, which contains a substantial portion of carbon-based nanoparticles (Wang et al., 2015a).

The type of NPs used can also influence their direct effect. Lamas et al. (2020) and Gangadoo et al. (2021) recently reviewed this topic and concluded that inorganic NP types (TiO_2_, Ag and SiO_2_) were the ones that exhibited a moderate to extensive impact on intestinal microbiota composition and activity. When using wax NPs, e.g. SLN given by gavage to rats in a first acute administration for 14 days, at dosages of 10 mg/kg, Bacteroides and *Bifidobacter*ium groups decreased (Madureira et al., 2016). The type of wax used to produce the SLN, either Witepsol or Carnauba, also resulted differently. An unpublished study also demonstrated that microbiota tend to adapt to the presence of the NPs, as no significant changes were observed were fed chronically for 6 weeks. SLN loaded with herbal extracts exhibited a prebiotic effect, promoting an increase in probiotic bacteria groups.

Also, calcium alginate beads of *Shewanella putrefaciens* Pdp11 changed the microbiota (ie. Proteobacteria) of gilthead seabream (*Sparus aurata L.*) fish specimens after 30 days of treatment with the absence of predominant bands related to Cyanobacteria and Vibrio genus and *Lactococcus* and *Lactobacillus* strains (Cordero et al., 2015a).

Animal models can be useful to evaluate several parameters; however, the conclusions regarding the effect of NPs size and daily dose were shown to be difficult to take. As previously mentioned at the beginning of this section, the animal studies are being avoided due to ethical concerns and lack of representative when comparing animals and human gut microbiota. Thus, more responsible studies need to be developed prior to perform studies within animal models. The most relevant *operands mode* found within the market it is to perform *in vitro* studies within human samples combined with environmental samples and run detection through PCR & NGS gathering information which enable the development of predictive digital tools based in machine learning. This approach enables to provide a powerful way to explore and better understand the NP and microbiota relationship, reducing the need for *in vivo* animal studies (McCoubrey et al., 2021). Nevertheless, these novel representative models that are arriving at the scientific field are in development and the relationship with the biochemical and haematological data and symptoms developed by the animals can also be correlated with microbiota modulation. This allows to predict the overall effects of NPs in the organism, in addition to toxicological effects that can only be observed *in vivo.*

## Conclusions and future perspectives

There is a great number of studies on NPs for food products applications, which contribute significantly, to better understand the route of NPs, especially for safety reasons and potential tissues accumulation effects. Throughout a product development process, it is important to evaluate the chemical and biological activity along the GIT tract. These studies can be first performed *in vitro*, to help the product development work, especially in terms of materials stability and resistance normal digestion conditions.

Before performing gut microbiota studies, it is important to evaluate the bio accessibility and bioavailability of NPs, comprehend their route throughout GIT, from mouth to colon, to assess chemical changes that occur during digestion and the effective quantities that reaches the intestine and will be available for absorption. The distinct stages of digestion, different pH and enzymes will lead to chemical and physical modifications that may originate alterations on NPs chemical and physical properties (agglomeration, dissolution etc.). A standardised model, which include all stages of digestion, such as INFOGEST protocol, might be the most adequate model to test NPs.

Studies concerning the interaction between different types of NPs and gut have been carried out to understand their impact and how to utilise their benefits towards human well-being and health promotion. The first approach is to test the effect of NPs on isolated microorganisms or representative species of gut microbiome. The microorganisms tested can be representative of beneficial bacteria or species linked to infectious diseases. A great part of these studies uses human faeces as substrates to perform fermentation trials and mimic the conditions prevailing at the colon. The use of faecal material enables the testing of several doses and different conditions, including the changes on the shape of bacteria.

After confirmation of toxicity using cell lines, animal models may be used. In fact, *in vivo* studies are very useful to investigate NPs applications. It allows to obtain biochemical data, follow the absorption and secretion paths of the encapsulated compound, and the presence of NPs in organs. Moreover, allows to collect the animal faecal material throughout the study and characterise the gut microbiota changes in terms of diversity and metabolic compounds produced. In addition, the antimicrobial activity and inhibition of a specific group, prebiotic activity, and enhancement of the growth of a certain microbiota group can be assessed. According to the literature, the dose and NPs physical format can affect gut microbiota, however in terms of size, there is not a specific trend. On the other hand, it is evident that dosage play an important role, as high doses originated great variations in microbiota and/or provoked pathological symptoms that can lead to microbiota dysbiosis. In addition, NPs may induce symptoms originated by microbiota dysbiosis. Inorganic NP (TiO_2_, Ag and SiO_2_) have been the most studied NPs and have consistently exhibited a moderate to extensive impact on intestinal microbiota composition and activity.

Further investigations should be carried out to improve the *in vitro* models and mimic as close as possible the *in vivo* conditions and hence avoid *in vivo* animal studies, which implicate the animal’s sacrifice. Our research group has optimised a continuous GIT process, which includes an absorption stage after gastric and intestinal digestion according to the INFOGEST protocol. After absorption, work on the interactions with blood cells and inclusion of other important cell lines to simulate other important organ tissues and predict the entire pharmacokinetic pathway of the metabolised molecules is also being carried out.
